# Pharmacological Evaluation of* Mentha spicata* L. and* Plantago major* L., Medicinal Plants Used to Treat Anxiety and Insomnia in Colombian Caribbean Coast

**DOI:** 10.1155/2018/5921514

**Published:** 2018-08-07

**Authors:** Daneiva C. Caro, David E. Rivera, Yanet Ocampo, Luis A. Franco, Rubén D. Salas

**Affiliations:** Biological Evaluation of Promising Substances Group, Faculty of Pharmaceutical Sciences, University of Cartagena, Cartagena 130015, Colombia

## Abstract

Anxiety disorders are highly prevalent, chronic, and disabling conditions that impose enormous health and economic costs both on individuals and on society. Medicinal plants are an invaluable source of bioactive metabolites that can be useful as new pharmacological treatment. Teas from* Mentha spicata* and* Plantago major* are employed by Colombian populations to treat stress and insomnia. This work was conducted to evaluate their anxiolytic and hypnotic properties. For this, we employed the Elevated Plus-Maze test and the sodium pentobarbital-induced hypnosis method using Wistar rats. Oral administration of* M. spicata* extract (1000 mg/Kg) significantly increased the exploration and time spent in the open arms, which indicates its anxiolytic activity. On the other hand, both* M. spicata* and* P. major* extracts (1000 mg/Kg) remarkably augmented the sleeping time induced by pentobarbital, suggesting a sedative and hypnotic effect of the plants extracts. In addition, the acute toxicological study demonstrated that the doses used did not induce mortality or toxicity effects at hepatic or renal level. The bioactivity seems to be related to several kinds of constituents, mainly phenolic compounds such as flavonoids and tannins. In conclusion, these results reinforce the potential use of these species in the therapy of anxiety.

## 1. Introduction

Within the disorders that affect the central nervous system (CNS), anxiety is one of the most frequently diagnosed conditions worldwide [1, 2]. Generalized anxiety disorder (GAD) is a well-defined condition characterized by excessive, uncontrollable, and persistent worry about everyday internal and external events. GAD is usually accompanied by psychological and somatic complaints, such as autonomic arousal, restlessness, fatigue, problems with concentrating, irritability, and sleep problems (insomnia, difficulty to fall or stay asleep, and poor quality sleep) [3, 4]. These clinical symptoms have a huge impact on individual's interpersonal relationships, work performance, and mental and physical health [5].

Although this disorder is especially common and impairing in high-income countries, the prevalence of GAD is rising globally, reaching public health significance in low- or middle-income countries, where investments in mental health are insufficient [2, 6]. In Colombia, GAD is a common mental disorder. Indeed, prevalence of anxiety has been on rising from 9.6% in 1993 to 19.3% in 2003 [7, 8]. Data from the National Mental Health Survey 2015 shows a higher prevalence for patients aged between 18 and 44 years, especially women, with a 12-month prevalence of 1.0% [2, 7].

Effective psychological and pharmacological treatments for anxiety disorders are available. Drugs therapies for GAD include benzodiazepines; antidepressants such as selective serotonin-reuptake inhibitors (SSRIs) and serotonin–noradrenaline-reuptake inhibitors (SNRIs); pregabalin; quetiapine; and buspirone [9]. In this regard, the use of benzodiazepines continues to be controversial. On one hand, unequivocal and robust evidence confirm their efficacy for GAD, both for short-term (e.g., of new-onset panic disorder) and long-term (e.g., of chronic GAD). On the other hand, concern about their extensive prescription and serious side effects ranges from respiratory, digestive, and immunological dysfunctions to deterioration of cognitive function, tolerance, physical dependence, and withdrawal syndrome [9, 10]. As a result of these limitations, the health and wellbeing of the patients and their families are affected. Therefore, new anxiety treatments are needed; yet since 2007, no new anxiolytics have been approved by the US Food and Drug Administration (FDA) [11].

In South American countries, including Colombia, GAD is also treated mainly with benzodiazepines such as diazepam, clonazepam, alprazolam, and lorazepam [12, 13]. However, plant-based drugs are popular since patients consider them as a natural, effective, and safe alternative to treat anxiety [14]. In fact, only 1.9% of Colombian patients visit the psychiatrist for GAD management, whereas 3.9% prefer to employ complementary and alternative medicine [8]. In the case of the Colombian Caribbean Coast, people often rely on the employment of herbs to treat stress and insomnia. Two popular teas are prepared with the leaves of* Mentha spicata* L. (spearmint), locally known as “yerba buena”, and* Plantago major* L. (plantain) locally known as “llantén”. Nevertheless, to date there is no scientific evidence supporting their effectiveness or safety.

Since research in the area of herbal psychopharmacology has revealed a variety of promising medicines that may provide benefit in the treatment of GAD [15, 16], this work evaluated the anxiolytic and hypnotic effects of the aqueous extract of the leaves of* M. spicata *and* P. major*, collected in Colombia.

## 2. Materials and Methods

### 2.1. Drugs and Chemicals

Diazepam (DZP; 10 mg/2 mL; VITECO S.A, Bogotá, Colombia) and sodium pentobarbital (SPT-Penthal; 64.8 mg/mL, Decno S.A.S., Bogotá, Colombia) were used as standard drugs. Saline solution (0.9% NaCl) was used as vehicle for drugs or test extracts. DZP, PTB, and extracts from* M. spicata* and* P. major* were prepared immediately before use and administered according to the experimental design described below.

### 2.2. Plant Material and Preparation of Total Extracts

Leaves of wild* M. spicata* and* P. major* were collected in Arjona, Bolívar (10°15′18′′ N -75°20′41′′ W), between July and August 2012. Taxonomic identification was performed by Biol. MSc. Felipe Alfonso Cardona Naranjo at Herbarium of the University of Antioquia, Colombia, and a voucher specimen of the whole plant was deposited (HUA 183.633 for* M. spicata* and HUA 183.634 for* P. major*). Extracts were prepared following the traditional methods of employment [17]. Fresh leaves of* P. major* or air-dried leaves of* M. spicata* were cleaned and crushed for extraction. In both cases, plant material (153.48 g* M. spicata* and 200 g* P. major*) was extracted with 3 L of hot distilled water at 60°C for 15 minutes. The obtained decoction was filtered, frozen at -80°C, and then lyophilized using FreeZone (Labconco, Kansas City, MO, USA). The lyophilized powder was stored at room temperature until experiments were performed.

### 2.3. Phytochemical Screening

In order to identify the secondary metabolites of the extracts, a phytochemical screening was carried out as previously described [18]. The presence of flavonoids, leucoanthocyanidins, phenolic compounds, triterpenes, steroids, quinones, alkaloids, tannins, and saponins was assessed.

### 2.4. Animals

Male Wistar rats, 2 months of age and 150-200 g of weight, provided by Instituto Nacional de Salud (Bogotá, Colombia), were used in the study. The animals were housed in filtered-capped polycarbonate cages in groups of five animals, fed with standard rodent food and water* ad libitum*, and maintained in a controlled environment with temperature at 24±2°C, relative humidity between 45 and 55%, and cycles of 12 h light/darkness. The experimental protocols were designed and conducted in accordance with the recommendations of the European Union regarding animal experimentation (Directive of the European Council 2010/63/EU) and were approved by the Committee of Ethics in Research of the University of Cartagena (Minute No. 63 of March 28, 2013). All the experiments were performed when the rats were in good health, normal activity, and in a randomized order [19].

### 2.5. Acute Toxicity Test

The safety of test extracts was evaluated using an acute oral toxicity test. As no previous toxicity information was available for both plants, a dosage level of 1000 mg/Kg was chosen to guarantee the occurrence of possible adverse effects during pharmacological evaluations. Briefly, rats were randomly distributed to groups (*n* = 5) and were treated orally with* M. spicata* extract,* P. major* extract, or vehicle by oral gavage (p.o.) using a feeding cannula. The animals were observed for general behavioral changes, signs of toxicity, and mortality continuously until 24 h after treatment, applying an Irwin test [20, 21]. Subsequently, animals were sacrificed by cervical dislocation and hepatic and renal samples were collected for histological analysis.

### 2.6. Histological Analysis

Samples were fixed in 10% buffered formalin, embedded in paraffin, sectioned, mounted onto slides, and stained with hematoxylin/eosin (H&E) according to standard protocols. The slides were analyzed by a blinded pathologist by light microscopy (Axio Lab.A1; Carl Zeiss, Thornwood, NY, USA). Slides from liver tissue were analyzed in terms of inflammation, necrosis, vascular congestion, regenerative changes, and abnormal growth. Signs of edema, tubulitis, regenerative changes, acute tubular necrosis, fibrosis, vascular congestion, and interstitial inflammatory infiltrate were examined in kidney samples.

### 2.7. Psychopharmacological Evaluation

#### 2.7.1. Elevated Plus-Maze Test (EPM)

The EPM was used to assess the anxiolytic activity of test extracts, following the method described by [22, 23], with some modifications. EPM apparatus is made of wood painted black and comprises two opposite closed arms (40 cm x 10 cm) with 40 cm high walls, crossed with two open arms of the same dimension. The four arms are connected by a central square (10 x 10 cm), forming a cross, and elevated to a distance of 50 cm from the floor. The animal behavior was recorded with a video camera located above the apparatus. For the experiment, rats were divided into four experimental groups (n=5) that received vehicle (control; saline 1 mL/Kg, p.o.);* M. spicata* extract (1000 mg/Kg, p.o.);* P. major* extract (1000 mg/Kg, p.o.); or DZP (1 mg/kg, i.p.). 30 minutes after the treatment, animals were placed individually at the intersection of the four arms of the EPM, facing the direction of one of the open arms and their behavior was recorded for 15 minutes. The entry to open/closed arms, as well as the cumulative time spent in open/closed arms, was recorded [24]. Entries were counted when an animal placed all four paws in an open or closed arm.

#### 2.7.2. Sodium Pentobarbital- (SPT-) Induced Hypnosis

To evaluate the sedative and hypnotic effect of test extracts, the SPT-induced sleeping time was employed. For this, rats were randomly divided into three groups (n=5) and treated with* M. spicata* extract (1000 mg/kg, p.o.),* P. major* extract (1000 mg/kg, p.o.), or vehicle (control; saline 1 mL/Kg, p.o.). After 30 minutes, SPT (30 mg/kg, i.p.) was administered and the animal was placed in an observation box. The latency to the onset of sedation and hypnosis (loss of righting reflex) and the sleeping time duration were recorded individually [25].

### 2.8. Statistical Analysis

Data are presented as mean ± standard error of the mean (S.E.M.) and analyzed by one-way analysis of variance (ANOVA), followed by Dunnett's post hoc test, to determine the differences between groups. Values of P<0.05 were considered significant.

## 3. Results

### 3.1. Extraction Yield and Phytochemical Screening

Total aqueous extracts from the leaves of* M. spicata* (yield 59.3%) and* P. major *(yield 15.6%) were obtained. Leaves were selected since infusions of these plants are used by the local population of Colombian Caribbean coast to treat anxiety, nervousness, and insomnia. Results of the phytochemical analysis are summarized in Table 1. Colorimetric assays revealed that both test extracts shared abundant presence of flavonoids, phenolic compounds, and tannins.

### 3.2. Acute Toxicity and Histological Analysis

**Figure 1:**
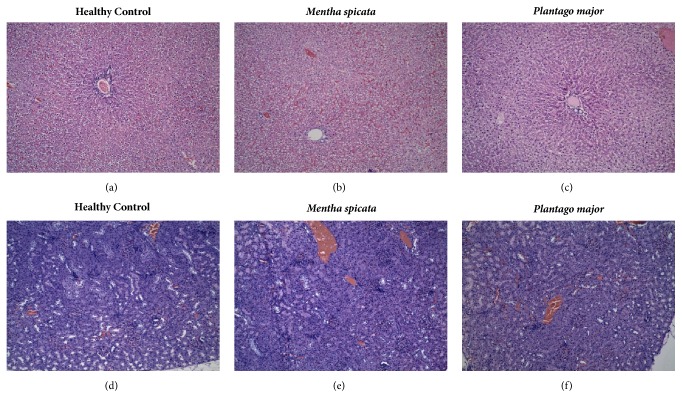
Toxicological evaluation of* M. spicata* and* P. major*. The histological analysis of liver (a–c) and kidney (d–f) samples from experimental animals was performed as described. Original magnification (10X). ((a) and (d)) Control group (vehicle-treated); ((b) and (e))* M. spicata* extract (1000 mg/Kg,* p.o.*); and ((c) and (f))* P. major* extract (1000 mg/Kg,* p.o.*).

Oral administration of test extracts (1000 mg/kg) did not cause lethality, changes in the normal behavior, or evidence of neurological alterations in rats, within 24 hours. At the time of sacrifice, all animals were well conditioned with normal physical appearance when compared to the control group (vehicle-treated). Consistently, necropsy did not reveal detectable abnormalities in rats treated with the extracts. With the exception of a slight increase of vascular congestion on liver tissue, the histological evaluation did not show important microscopic disturbances on liver or kidney that could be related to the administration of* M. spicata* or* P. major* extracts (see Figure 1).

### 3.3. Elevated Plus-Maze Test

**Figure 2:**
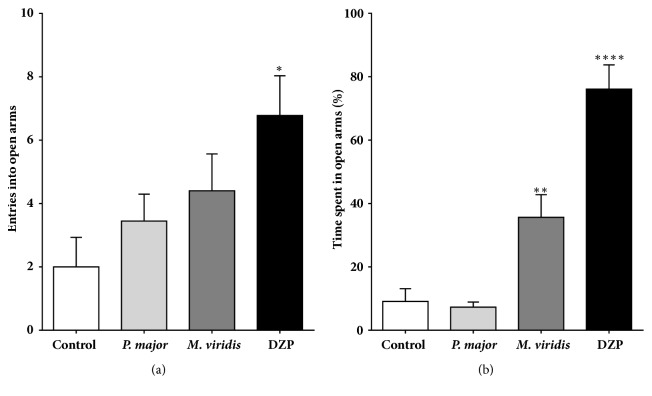
Evaluation of the anxiolytic effect of the aqueous extract obtained from* M. spicata* and* P. major*. The influence of* M. spicata* (1000 mg/Kg,* p.o.*);* P. major* (1000 mg/Kg,* p.o.*); and diazepam (DZP, 1 mg/Kg,* i.p.*) was assessed by the entries into open arms (a) and the percentage of time spent in the open arms (b) of the Elevated Plus-Maze (EPM) apparatus. Results are expressed as mean ± S.E.M. (*n*=10). *∗∗∗∗*P<0.0001, *∗∗*P<0.01, and *∗*P<0.05 ANOVA are significantly different compared with the control group.

As shown in Figure 2, the administration of* M. spicata* extract (1000 mg/Kg, p.o.) significantly increased the amount of time spent in the open arms of the EPM, when compared to vehicle-treated rats (9.08 versus 35.62%, P<0.01). Although not significant,* M. spicata*-treated mice also tended to move into the open arms with more frequency. In contrast, oral treatment with* P. major* extract (1000 mg/Kg) did not produce any effect on animal's behavior. DZP (1 mg/Kg, i.p.), as expected, showed a significant increased number of entries and time spent in the open arms, as compared with control group (P<0.0001).

### 3.4. Pentobarbital-Induced Hypnosis Test

**Figure 3:**
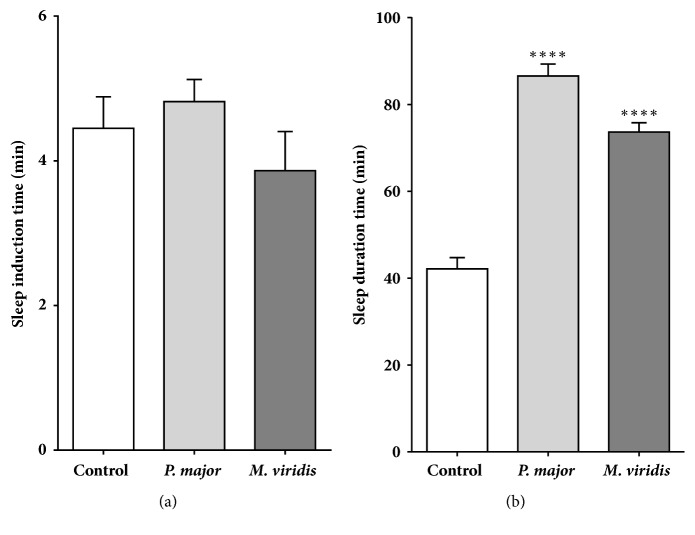
Hypnotic effect of the aqueous extract obtained from* M. spicata* and* P. major*. The induction (a) and duration (b) of total sleeping time induced by sodium pentobarbital (SPT, 30 mg/kg,* i.p.*) were calculated for Wistar rats previously treated with vehicle (saline);* M. spicata* (1000 mg/Kg,* p.o.*); and* P. major* (1000 mg/Kg,* p.o.*). Results are expressed as mean ± S.E.M. (*n*=5). *∗∗∗∗P*<0.0001 ANOVA is significantly different compared with the control group.

None of the tested extracts modified the onset to sedation and hypnosis induced by SPT (see Figure 3(a)). Conversely,* M. spicata* and* P. major* extracts (1000 mg/Kg, p.o.) significantly prolonged the sleeping time in SPT-treated mice, from 42.13±2.62 min to 73.64±2.16 min and 86.57±2.75 min, respectively (see Figure 3(b)).

## 4. Discussion

The Colombian Caribbean coastal environments are extremely varied, ranging from high mountains to low beach ridges, mangrove swamps typical of small- to medium-sized river deltas, and desert areas [26]. This is translated into an appealing landscape, attractive to national and foreign visitors, as well as a unique flora and fauna. In this region, some wild-grown medicinal plants are still employed frequently, especially as a replacement of ineffective conventional drugs. In this work, we demonstrated the anxiolytic and hypnotic effect of* Mentha spicata* and* Plantago major* extracts. To our knowledge, this study constitutes the first report of the psychopharmacological effect of these plants.

The employment of* M. spicata* and* P. major* to treat nervous irritability and insomnia is not limited to Colombian Caribbean region. In fact, these plant species have been used for centuries as sedative or to treat anxiety and nervous affections (insomnia, stress, and agitation) throughout Europe, Asia, and America [27–30]. Furthermore,* M. spicata* and* P. major* are used for different purposes in folk medicine all over the world.* M. spicata* is employed to treat gastrointestinal complaints (stomach ache, flatulence, indigestion, and nausea) [17, 31, 32]. Similarly,* P. major* is used as laxative, diuretic, antihemorrhagic, wound-healer, expectorant, and anti-inflammatory [33–35]. Despite their extended medicinal employment, there are few chemical, pharmacological, or toxicological studies regarding the bioactivity of both species.

Our study initiated with the production of a standard extract of* M. spicata* and* P. major* leaves, that enabled us to replicate the traditional preparation, guarantee the extraction of the bioactive metabolites, and administer a fixed dose to rats. For this, we prepared both extracts following the directions of a handbook published by the Program for Applied Research and Diffusion of Traditional Plant Uses in the Caribbean (TRAMIL) [17]. Decoction of both plants yielded a good amount of extracts that allowed the administration of a dosage level (1000 mg/Kg, p.o.) with some resemblance to the human daily consumption in the Caribbean (1 cup/day; 3-10 g of leaves) to treat nervousness.

Taking into account the fact that we employed a relatively high dosage level and that previous studies reported toxic and adverse effects related to prolonged consumption of* M. spicata* (hepatic, renal and hormonal problems in rats and humans) and* P. major* (laxative and hypotensive effect in humans), we decided to evaluate the acute toxicity of extracts [36–39]. Our findings revealed that acute treatment with aqueous extracts of both plants do not induce mortality or macroscopic signs of toxicity. Complementarily, histologic analysis did not cause important changes in kidney or liver tissue, with exception of a minor increase of vascular congestion in the liver of rats treated with both extracts. Although these results indicate the safety of test extracts during the pharmacological evaluation performed by us, further studies involving chronic administration of the standardized extracts are mandatory, especially when local communities rely on these plants as a safe strategy to control their anxiety.

The bioactivity of* M. spicata* and* P. major* on the central nervous system was evaluated using two experimental models: Elevated Plus-Maze (EPM) and pentobarbital-induced hypnosis. The EPM test revealed a remarkable anxiolytic activity of* M. spicata*, reflected by a significant increase of the percentage of time in the open arms of the apparatus, a reliable measurement of lower levels of stress [40, 41]. Indeed, among approach-avoidance conflicting tests, EPM is one of the most popular since it is considered to model closely general anxiety disorders and specific phobias, largely based on their perceived face validity and sensitivity to benzodiazepine anxiolytics [42]. Moreover,* M. spicata* extract showed a hypnotic effect, reflected by a significant prolongation of sleeping time induced by SPT, an indicative of CNS-depressant activity. With regard to* P. major*, we found that the oral administration of this extract significantly enhanced the duration of the SPT-induced hypnotic effects, without exerting important anxiolytic effects. Therefore, the bioactivity described in this work for* M. spicata* and* P. major* is according to their employment in traditional medicine.

On the other hand, the phytochemical analysis revealed that extracts from* M. spicata* and* P. major* shared the presence of phenolic compounds like flavonoids and tannins. Interestingly, plant-derived flavonoids are CNS-active molecules with elevated anxiolytic potency that appear to modulate *γ*-amino butyric acid-A (GABA_A_) receptor function [43–45]. Thus, our work suggests that natural flavonoids from* M. spicata* and* P. major* might be attractive leads to develop new drugs to treat anxiety. Nevertheless, the chemical composition of both extracts should be carefully studied to verify if a single or a mixture of metabolites are responsible for their bioactivity.

## 5. Conclusions

This work reinforces the potential use of* M. spicata* and* P. major* in the therapy of anxiety demonstrating that aqueous extracts from these species induced a sedative response in rats that was reflected in anxiolytic and hypnotic effects. In addition, the acute toxicological study demonstrated that the doses used in this work did not induce mortality or adverse effects at hepatic or renal level. In short, our findings in rats are in accordance with the effects described for* M. spicata* and* P. major* in Colombian traditional medicine, which is employed as a sedative agent. Thus, further studies to isolate new naturally occurring metabolites useful to treat CNS disorders are warranted.

## Figures and Tables

**Table 1:** Preliminary phytochemical screening of aqueous total extract obtained from *M. spicata* and *P. major* leaves.

Metabolite	*M. spicata*	*P. major*
Flavonoids	**++**	**++**
Leucoanthocyanidins	-	-
Phenolic compounds	**+++**	**+++**
Steroids and/or triterpenes	-	-
Quinones	-	-
Alkaloids	-	-
Tannins	**+++**	**+++**
Saponins	-	-

Test results are classified as strongly positive (+++), positive (++), weakly positive (+), and non-detected (−).

## Data Availability

The data used to support the findings of this study are included within the article.

## References

[1] Baxter A. J., Vos T., Scott K. M., Ferrari A. J., Whiteford H. A. (2014). The global burden of anxiety disorders in 2010. *Psychological Medicine*.

[2] Ruscio A. M., Hallion L. S., Lim C. C. (2017). Cross-sectional Comparison of the Epidemiology of. *JAMA Psychiatry*.

[3] American Psychiatric Association (2013). *Diagnostic and Statistical Manual of Mental Disorders*.

[4] Cuijpers P., Sijbrandij M., Koole S., Huibers M., Berking M., Andersson G. (2014). Psychological treatment of generalized anxiety disorder: A meta-analysis. *Clinical Psychology Review*.

[5] Generoso M. B., Trevizol A. P., Kasper S., Cho H. J., Cordeiro Q., Shiozawa P. (2017). Pregabalin for generalized anxiety disorder: An updated systematic review and meta-analysis. *International Clinical Psychopharmacology*.

[6] Chisholm D., Sweeny K., Sheehan P. (2016). Scaling-up treatment of depression and anxiety: A global return on investment analysis. *The Lancet Psychiatry*.

[7] MINSALUD. “Encuesta Nacional de Salud Mental 2015”, ed. Javegraf, Bogota, Colombia, 2016

[8] Posada-Villa MD, Buitrago-Bonilla TS, Medina-Barreto TS, Rodríguez-Ospina. MD M. (2006). Trastornos de ansiedad según distribución por edad, género, variaciones por regiones, edad de aparición, uso de servicios, estado civil y funcionamiento/discapacidad según el Estudio Nacional de Salud Mental-Colombia. *NOVA*.

[9] Craske M. G., Stein M. B. (2016). Anxiety. *The Lancet*.

[10] Lader M., Kyriacou A. (2016). Withdrawing Benzodiazepines in Patients With Anxiety Disorders. *Current Psychiatry Reports*.

[11] Stein M. B., Craske M. G. (2017). Treating anxiety in 2017 optimizing care to improve outcomes. *Journal of the American Medical Association*.

[12] Gómez S., León T., Macuer M., Alves M., Ruiz S. (2017). Benzodiazepine use in elderly population in latin America. *Revista Médica de Chile*.

[13] Machado-Alba J. E., Alzate-Carvajal V., Jimenez-Canizales C. E. (2015). Trends in the consumption of anxiolytic and hypnotic drugs in a Colombian population. *Revista Colombiana de Psiquiatria*.

[14] Voces García D., Díaz Gómez Calcerrada C., Puente García N. (2002). Uso de plantas medicinales en el tratamiento de la ansiedad y la depresión. *FMC - Formación Médica Continuada en Atención Primaria*.

[15] Sarris J., McIntyre E., Camfield D. A. (2013). Plant-based medicines for anxiety disorders, part 1: a review of preclinical studies. *CNS Drugs*.

[16] Sarris J., McIntyre E., Camfield D. A. (2013). Erratum: Plant-based medicines for anxiety disorders, Part 2: A review of clinical studies with supporting preclinical evidence (CNS Drugs (2013) 27:4 (301-319) DOI:10.1007/s40263-013-0059-9). *CNS Drugs*.

[17] TRAMIL (2008). *Plantas medicinales caribeñas para la atención primaria, Manual Práctico*.

[18] Herrera Herrera Alejandra, Franco Ospina Luis, Fang Luis, Díaz Caballero Antonio (2014). Susceptibility of *Porphyromonas gingivalis* and *Streptococcus mutans* to Antibacterial Effect from *Mammea americana*. *Advances in Pharmacological Sciences*.

[19] Taiwo A. E., Leite F. B., Lucena G. M. (2012). Anxiolytic and antidepressant-like effects of Melissa officinalis (lemon balm) extract in rats: Influence of administration and gender. *Indian Journal of Pharmacology*.

[20] CYTED (1995). *Manual de Técnicas de Investigación, Subprograma X. Química Fina Farmacéutica. Proyecto X-1. Búsqueda de Principios Bioactivos en Plantas de la Región*.

[21] Vergel N. (2011). Estudio de la actividad anticonvulsivante de metabolitos secundarios tipo cumarina [Tesis de Doctorado]. *Biblioteca virtual para la vigilancia en salud pública de Colombia*.

[22] Pellow S., Chopin P., File S. E., Briley M. (1985). Validation of open: closed arm entries in an elevated plus-maze as a measure of anxiety in the rat. *Journal of Neuroscience Methods*.

[23] Pellow S., File S. E. (1986). Anxiolytic and anxiogenic drug effects on exploratory activity in an elevated plus-maze: A novel test of anxiety in the rat. *Pharmacology Biochemistry & Behavior*.

[24] Walf A. A., Frye C. A. (2007). The use of the elevated plus maze as an assay of anxiety-related behavior in rodents. *Nature Protocols*.

[25] Sugden D. (1983). Psychopharmacological effects of melatonin in mouse and rat. *The Journal of Pharmacology and Experimental Therapeutics*.

[26] Correa I. D., Alcantara-Carrio J., Gonzalez D. A. (2005). Historical and Recent Shore Erosion along the Colombian Caribbean Coast , Journal of Coastal Research. *special*.

[27] Blumenthal M., Cavaliere C., Rea P., Lynch M. (2010). Growth and market trends for herbal products in the United States. *Planta Medica*.

[28] Naghibi F., Mosaddegh M., Mohammadi Motamed M., Ghorbani A. (2005). Labiatae Family in folk Medicine in Iran: from Ethnobotany to Pharmacology. *Iranian Journal of Pharmaceutical Research*.

[29] Chandra S., Rawat D., Chandra D., Rastogi J. (2016). Nativity, Phytochemistry, Ethnobotany and Pharmacology of Dianthus caryophyllus. *Research Journal of Medicinal Plant*.

[30] Yeung W.-F., Chung K.-F., Man-Ki Poon M. (2012). Chinese herbal medicine for insomnia: a systematic review of randomized controlled trials. *Sleep Medicine Reviews*.

[31] Loera J. A., Black S. A., Markides K. S., Espino D. V., Goodwin J. S. (2001). The use of herbal medicine by older Mexican Americans. *The Journals of Gerontology. Series A, Biological Sciences and Medical Sciences*.

[32] Ahmed H. M. (2016). Ethnopharmacobotanical study on the medicinal plants used by herbalists in Sulaymaniyah Province, Kurdistan, Iraq. *Journal of Ethnobiology and Ethnomedicine*.

[33] Fonnegra G. R., Jimenez R. S., 2. (2007). Plantas medicinales aprobadas en Colombia.

[34] Kültür Ş. (2007). Medicinal plants used in Kırklareli Province (Turkey). *Journal of Ethnopharmacology*.

[35] Neves J. M., Matos C., Moutinho C., Queiroz G., Gomes L. R. (2009). Ethnopharmacological notes about ancient uses of medicinal plants in Trás-os-Montes (northern of Portugal). *Journal of Ethnopharmacology*.

[36] Capasso R., Izzo A. A., Pinto L., Bifulco T., Vitobello C., Mascolo N. (2000). Phytotherapy and quality of herbal medicines. *Fitoterapia*.

[37] Akdogan M., Kwiwnc I., Oncu M., Karaoz E., Delibas N. (2003). Investigation of biochemical and histopathological effects of Mentha piperita L. and Mentha spicata L. on kidney tissue in rats. *Human & Experimental Toxicology*.

[38] Akdogan M., Ozguner M., Aydin G., Gokalp O. (2004). Investigation of biochemical and histopathological effects of Mentha piperita Labiatae and Mentha spicata Labiatae on liver tissue in rats. *Human & Experimental Toxicology*.

[39] Akdogan M., Ozguner M., Kocak A., Oncu M., Cicek E. (2004). Effects of peppermint teas on plasma testosterone, follicle-stimulating hormone, and luteinizing hormone levels and testicular tissue in rats. *Urology*.

[40] Cruz A. P. M., Frei F., Graeff F. G. (1994). Ethopharmacological analysis of rat behavior on the elevated plus-maze. *Pharmacology Biochemistry & Behavior*.

[41] Espejo E. F. (1997). Structure of the mouse behaviour on the elevated plus-maze test of anxiety. *Behavioural Brain Research*.

[42] Griebel G., Holmes A. (2013). 50 years of hurdles and hope in anxiolytic drug discovery. *Nature Reviews Drug Discovery*.

[43] Paladini A. C., Marder M., Viola H., Wolfman C., Wasowski C., Medina J. H. (1999). Flavonoids and the central nervous system: From forgotten factors to potent anxiolytic compounds. *Journal of Pharmacy and Pharmacology*.

[44] Hanrahan J. R., Chebib M., Johnston G. A. R. (2011). Flavonoid modulation of GABAA receptors. *British Journal of Pharmacology*.

[45] Hanrahan J. R., Chebib M., Johnston G. A. R. (2015). Interactions of flavonoids with ionotropic GABA receptors. *Advances in Pharmacology*.

